# Importance of “meal first” strategy and effective situations of supplement use in elite athletes: Japan high performance sport center position stand

**DOI:** 10.3389/fspor.2023.1188224

**Published:** 2023-06-13

**Authors:** Jun Yasuda, Kanae Myoenzono, Eri Takai, Makiko Toguchi, Shiori Tsunezumi, Chika Kondo, Aya Kaizaki, Shoko Ode, Hiroka Ohno, Keiko Namma-Motonaga, Akiko Kamei

**Affiliations:** Japan High Performance Sport Center, Japan Institute of Sport Sciences, Tokyo, Japan

**Keywords:** meal first, Olympic, Paralympic, food, ergogenic aid, doping, mastication, education

## Abstract

The “meal first” strategy is traditionally recommended for athletes' conditioning. However, the importance of the “meal first” principle has not been detailly well documented in athletes' lives. Supplement use has recently become a common part of athletes' diets, but unmonitored supplement use can cause negative consequences, such as anti-doping violations and health issues. Therefore, this review summarizes how the “meal first” strategy and planned supplement use are important for enhancing athletes’ health and performance. We believe that the “meal first” strategy is beneficial in terms of the following aspects: (1) consumption of multi-nutrients and other functional components simultaneously; (2) positive effects on psychological well-being; (3) contribution to athletes' health by way of mastication; and (4) less risk for anti-doping violations. Before supplement use, we recommend that athletes first verify their basic factors (e.g., diet, training, and sleep), given that the benefits of supplements are examined and demonstrated with the control of those factors. Otherwise, athletes cannot obtain maximal benefits from the supplements. In contrast, there are situations in which supplements in athletes' lives can be advantageous, such as (1) nutrient deficiency due to ongoing dietary characteristics; (2) interruption of meals due to disease; (3) inaccessibility of quality food during athletic travel; (4) difficulty preparing food due to societal restrictions associated with disasters or infection outbreaks; (5) having a meal before, during, or after exercise is difficult; and (6) achieving targeted intake of performance-enhancing ingredients is not practical. In summary, we emphasize that the “meal first” strategy is recommended for athletes' conditioning, but there are several contexts when supplement use can be more useful in athletes' lives.

## 1. Introduction

Although there is no agreed-upon definition of a supplement when it comes to dietary additives, we can learn from the simplistic viewpoint offered by Oxford Learner's Dictionary, which is that a supplement is “a thing that is added to something else to improve or complete it” (1). From this perspective, we can regard supplements for athletes as items added to basic factors (i.e., diet, exercise, or sleep) for achieving enhanced performance. The Japan High Performance Sport Center (HPSC) has a research department known as the Japan Institute of Sport Sciences, which designates supplements according to the following two tenets from the International Olympic Committee (IOC) consensus statement (2). First, dietary supplements are products that are intentionally consumed above and beyond the habitual diet with the aim of preventing a nutrient deficiency. Second, performance supplements are products that are intentionally consumed with the aim of enhancing one's athletic performance. Sports foods, including products that provide nutrients and energy in more convenient forms (e.g., drinks, gels, and bars) compared to traditional supplements or meal replacements, are becoming more common both in and beyond sporting events. In this review, we define dietary supplements, performance supplements, and sports foods as supplements.

A high percentage of supplement use among elite athletes has been reported (3). For example, 70% of Canadian athletes at the Sydney 2000 Olympic Games (4) and more than 90% of Japanese athletes (including candidates) at the Rio 2016 Olympic Games used supplements (5). However, several expert consensus statements emphasize that athletes should first confirm through dietary assessment by a sports-trained dietitian or nutritionist whether targeted nutrients or components are actually consumable from regular meals before resorting to supplementation (2, 6, 7). This is because supplements should be an additional option to a proper diet, one of the basic factors for promoting athletes' overall conditioning. Furthermore, supplement use is frequently accompanied by anti-doping violations (8). While the cause of intentional doping is obvious, unintentional doping is caused by invisible routes, such as contamination from manufacturing processes (9), misleading information on labels (10), or missing information about prohibited substances on labels (10, 11). Thus, avoiding unintentional doping is almost impossible, even if athletes pay extreme attention to the use of supplements. Unintentional doping is confirmed annually in Japan (12). To our knowledge, there are no studies investigating whether athletes seek professional confirmation of their dietary status and need before using supplements. Not only does this raise the potential for elite athletes to cause anti-doping violations and overdose of nutrients or components, but it is also likely that athletes will not obtain the expected supplement benefits anyway. Therefore, we underscore the importance of strengthening the basic factors underlying enhanced performance and caution against supplement use for athletes without proper education. This review aims to address the current gap in understanding safe and effective supplement use among athletes. We provide evidence-based guidance to reduce risks and enhance benefits, thereby promoting informed, responsible supplement use in sports.

In this review, recognizing the emerging importance and prevalent use of supplements among athletes and the associated risks, we aim to provide a comprehensive, up-to-date resource to support the improvement of athletes' health and performance. We summarize the following elements: (1) the reason why “meal first” is recommended, emphasizing the importance of a balanced diet before resorting to supplementation; (2) the importance of strengthening basic factors such as diet, exercise, and sleep, which is often overlooked due to the reliance on supplementation; (3) the effective contexts and precautions for supplement use, shedding light on the knowledge gap in this area; and 4) the anti-doping strategy, highlighting the importance of being vigilant about unintentional doping from supplement use, as the HPSC position stand.

## 2. Methodology

We usually support Japanese elite athletes—candidates for the Olympic and Paralympic games and counsel them on the best nutritional strategy with consideration of their states of practice, training, and rehabilitation. In this capacity, we often encounter issues related to their dietary behavior and supplement use. Consequently, we have compiled insights from these interactions and elected to articulate our position stand firmly grounded in scientific literature. The search for records was carried out through the official websites of relevant organizations (World Health Organization (WHO), World Anti-Doping Agency (WADA), Food and Drug Administration in the United States (FDA), The U.S. Anti-Doping Agency (USADA), Japan Anti-Doping Agency (JADA), and Japan Chemical Analysis Center (JCAC)), and database (PubMed). We used keywords related to our topic, including “doping,” “food,” “meal,” “diet,” “supplement,” “athlete,” “food hygiene,” and their combinations. Records were selected based on their relevance to the topic of meal-first strategy and anti-doping in sports. The initial selection was based on the review of titles and abstracts, followed by a full-text review. During this process, we discussed whether the reviewed articles were appropriate for the topic of this study with sports doctors and pharmacists in the HPSC who take care of the Olympic and Paralympic athletes.

## 3. The reason why “meal first” is recommended

**Figure 1:**
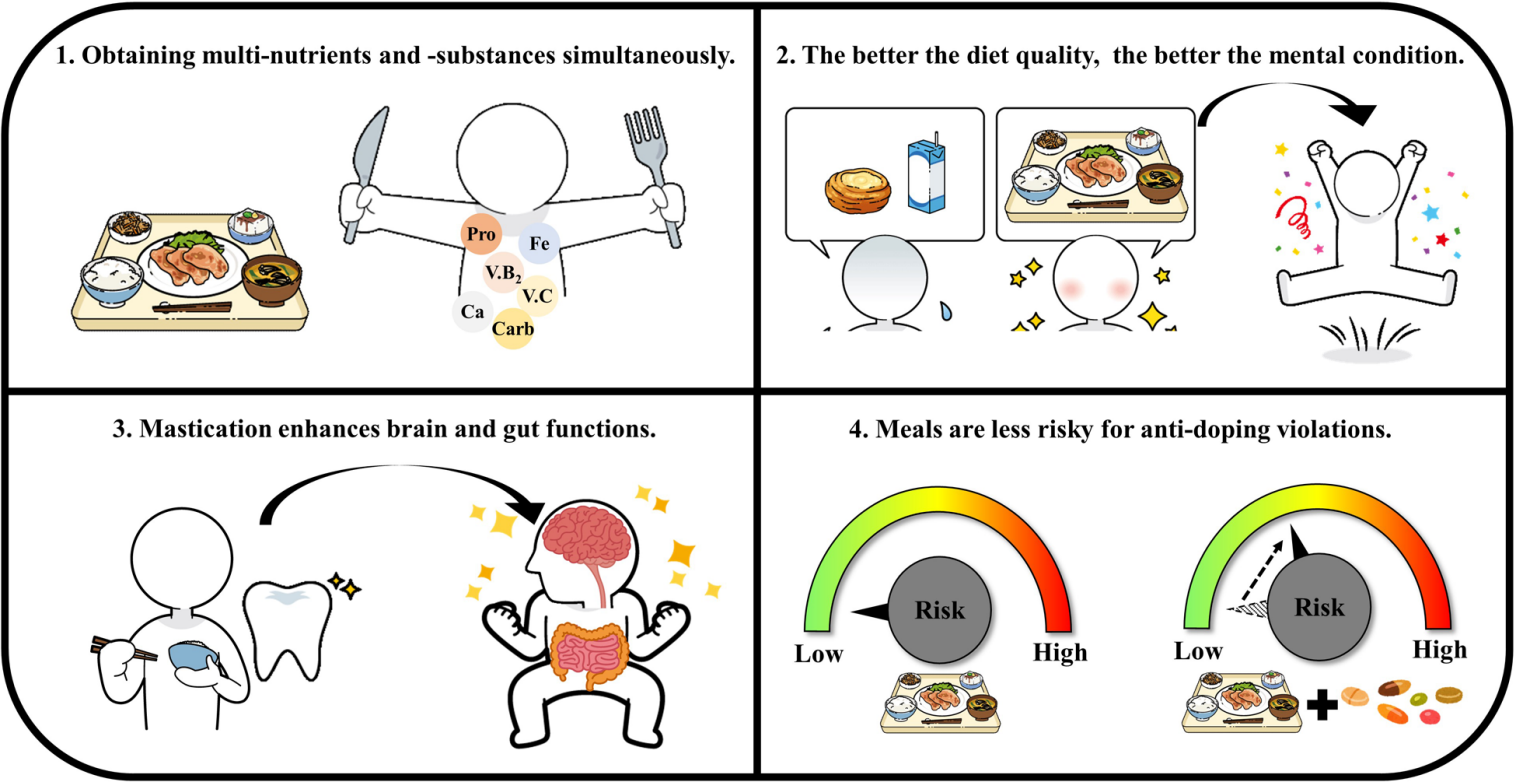
Four perspectives of the HPSC in recommending the “meal first” strategy.

Recently, the term “food first” was stated in brilliant reviews by Maughan's group (13) and the UEFA expert group (7). However, in Japan, the word “food” encompasses a broad range of items, including supplements and products made with supplements. Therefore, using the phrase “food first” could lead to confusion among athletes and their support networks. Even though we respect the “food first” theory, the HPSC thinks that the phrase “meal first” is more appropriate in Japan to empower athletes' conditioning based on four perspectives (Figure 1). First, meals are useful for obtaining macronutrients and micronutrients, fiber, polyphenols, and other active substances (14) simultaneously. Second, meals can positively impact one's psychological status (e.g., feelings and mood) (15). In fact, increasing the quality of meals (e.g., increasing the intake of fruits, vegetables, fish, whole grains, legumes, and olive oil) has been reported to improve depression and anxiety (16). More frequent eating with family and not eating alone was also reported to be associated with better emotional well-being (17). Thus, we believe that preparing meals and also meal occasions is effective for conditioning the psychological status of athletes. Third, meals require more mastication to consume and absorb than ready-to-eat supplements. However, to our knowledge, there is no study on the psychological effects of mastication in athletes, though it may contribute to the improvement of athletes' health. For older populations, mastication has been reported to influence food choice (18) and even cognitive functioning (19). In addition, meals accompanied by mastication tend to contain many important food residues, such as fiber, which is beneficial for the gut microbiome (20). Fourth, we believe that meals are less risky than supplements for anti-doping violations (10, 11, 21, 22) because anti-doping violations related to regular meal consumption have been confirmed in very limited areas only (23) (details in the Anti-doping section).

## 4. The importance of strengthening basis factors (diet, exercise, and sleep) before supplement use

**Figure 2:**
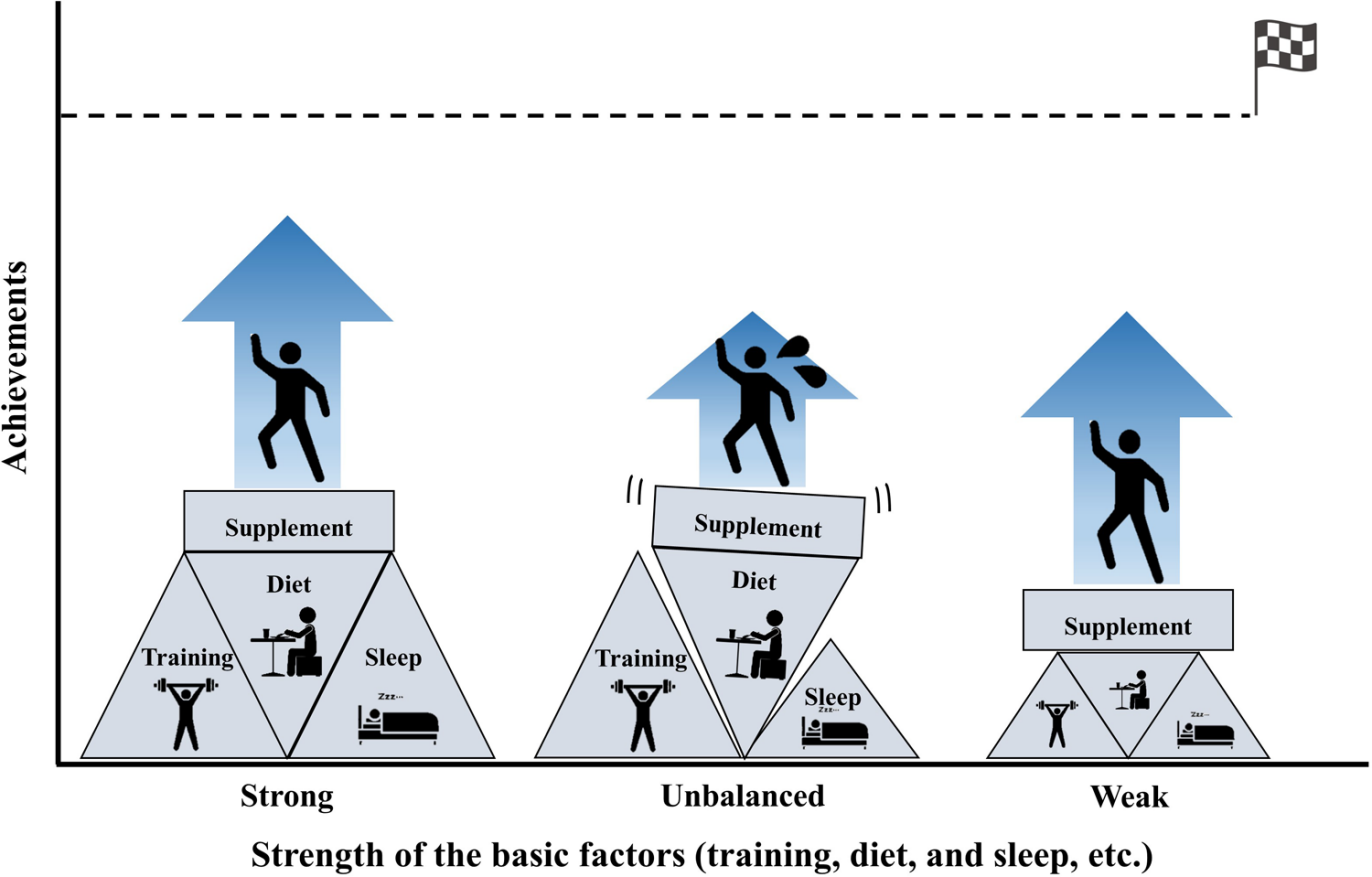
Importance of strengthening basic factors before turning to supplement use.

In sports, supplements can provide additional benefits when athletes' basic factors (diet, training, sleep, etc.) are already balanced and strengthened (Figure 2). In other words, athletes cannot achieve the expected benefits from supplements when the basic factors are not controlled; moreover, the risk of anti-doping violations increases with an increasing number of supplements. Although this simple system seems straightforward, we often observe situations where athletes, coaches, and trainers prioritize the use of supplements over basic factors. This suggests a prevailing belief that supplements possess extraordinary abilities to enhance an athlete's condition.

**Figure 3:**
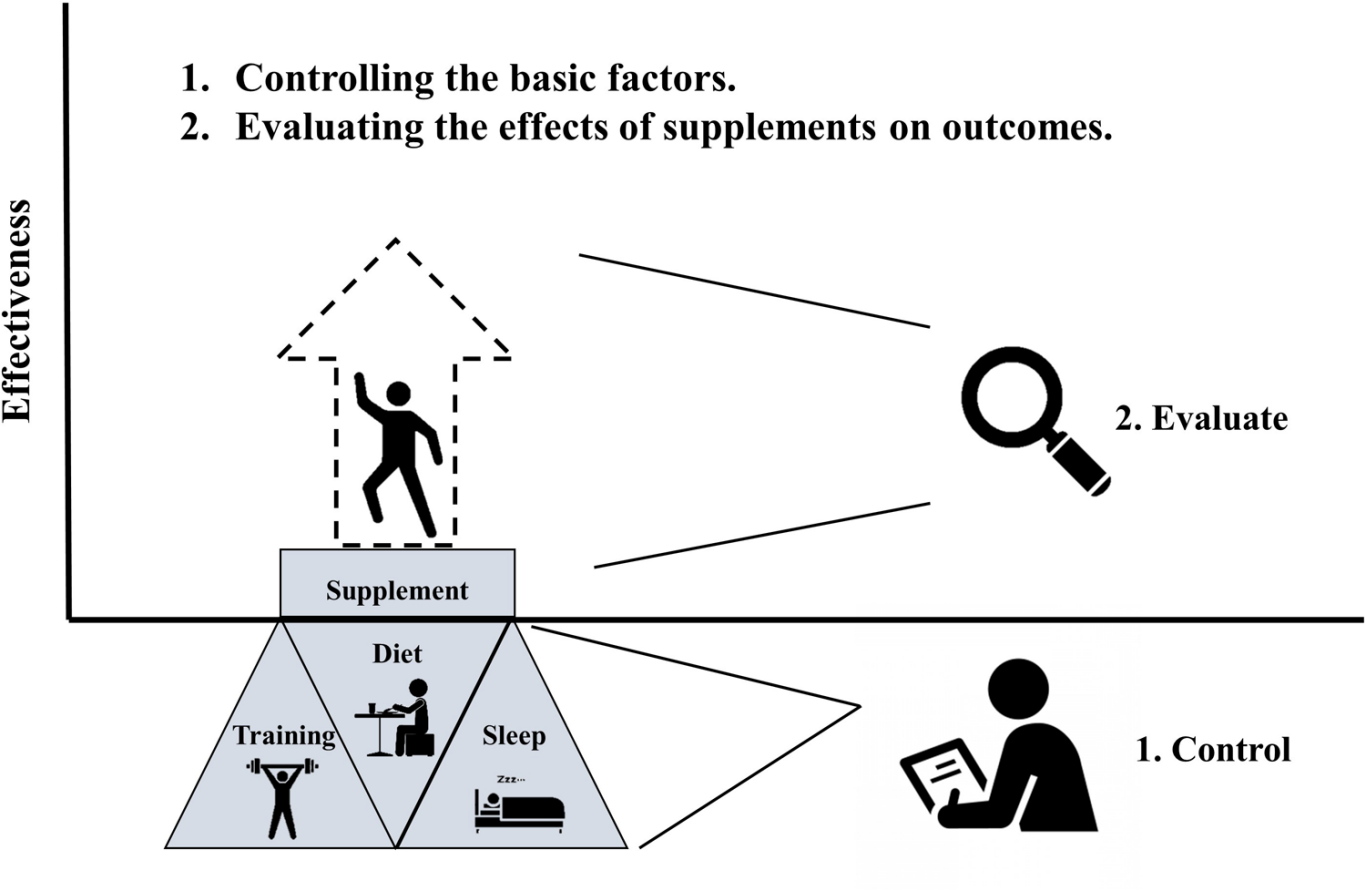
Characteristic of research examining the effects of supplements on outcomes.

Likewise, athletes and their professional points of contact (e.g., coaches, trainers, and dietitians) need to understand the methodological characteristics of studies examining the effects of supplements (24). These studies first control for basic factors, then evaluate the pure effects of supplements on performance/health-related outcomes (Figure 3). Without statistically controlling for baseline individual differences in diet, training, and sleep, studies would be unable to clearly evaluate the independent effects of supplements on key outcomes of interest. Lay consumers of the scientific literature need to understand that the gains attributed to supplements may not represent their own potential for gain if their basic diet, training, and sleep are not already maximized.

## 5. The effective contexts and precautions for supplement use

### 5.1. The effective contexts for supplement use

We believe that there are several situations in which careful supplement use can be more effective than adopting the “meal first” strategy. These are summarized in Table 1, although it should be mentioned that other situations may exist. We need to confirm whether supplement use in these contexts is the best method to support athletes while considering suitability, practicality, and convenience.

**Table 1:** Effective situations for supplement use.

Situations	Notes
1. Some nutrients are deficient due to characteristics of the diet.	Athletes can become nutrient-deficient for several reasons, such as weight loss, food allergy, intolerance (e.g., lactose or gluten) (25), food preference, and dietary styles (e.g., vegan or vegetarian) (26). In these situations, supplements can be effective for preventing or countering expected deficiencies.
2. Having meals is difficult due to a disease process.	Respiratory disease (mainly upper respiratory tract diseases) is reported as the most common disease in athletes (27). The incidence rate of respiratory disease in elite athlete populations has been reported to be higher compared to that of the general population (28). The higher incident rate in elite athletes can be connected to a reduction in immune system functioning by high-intensity exercise (29–31) or overseas expeditions (32–34). For a summary, see the latest review (35). Furthermore, studies have confirmed that appetite (36) and food intake were reduced during disease (37). The HPSC group is concerned that athletes, who tend to be more active than the general population, are more prone to severe sickness.
3. Accessibility to quality foods is difficult during athletic travel.	Many elite athletes experience situations in which they travel abroad for practice and competition. If the habitual foods and the food hygiene of the places where they stay are not organized, deficiencies of nutrients or even illness and infections may occur due to the limited availability of food options (38). Thus, the HPSC group believes that it is important to investigate the food environment of athletics venues and prepare supplements that can be effective for the maintenance of athletes’ conditions before traveling abroad.
4. Preparing food can be restricted by external situations such as disasters or outbreaks of infection.	In countries where disasters (e.g., tsunami or earthquake) frequently occur, the pathway to preparing foods is often shut down. Similarly, due to infections such as COVID-19, certain geographic areas can be locked down, closing off entire food pathways. For example, Japan frequently experiences disasters in a given year. When food pathways are closed in Japan, carbohydrate-based foods are provided to refugees (39) mainly in the form of rescue energy intake (40). This situation can negatively impact an athlete's body composition (41), as nutrients other than carbohydrates are needed to prevent deconditioning. Compared to conventional foods, supplements as a stockpile may be more useful in this situation since supplements usually have less water content and less chance of food spoilage (42).
5. Having a meal before, during, and after exercise is difficult.	When meal timing is restricted due to the characteristics of the sport, supplement use may be more beneficial than meals. For example, athletes engaged in snow sports spend most of their time on mountains for practices and competitions. In this scenario, supplement use is useful as it facilitates easy and convenient acquisition of necessary nutrients and components. In addition, studies have confirmed that appetite was reduced after both aerobic (43–46) and resistance (47, 48) exercises. To promote recovery between practices or competitions, supplements that have faster digestibility (49) are more effective for selectively obtaining target nutrients compared to meals. Yet, at the same time, the reduction in appetite recovers to that of resting baseline within 60-min after exercise (44, 47, 50). Thus, the “meal first” strategy, which can provide multiple nutrients to a body simultaneously, is recommended if prompt recovery is not necessary.
6. Achieving targeted intake of components to enhance performance is not practical.	Achieving the targeted intake of performance-enhancing components [e.g., caffeine, creatine, nitrate, *β*-alanine, and sodium bicarbonate (13)] may be difficult only from meals or whole foods. For example, several systematic reviews have reported that consuming 3 mg/kg weight of caffeine at least 60-min before exercise is beneficial to enhance performance (51–53). If the weight of athlete is 80 kg, the targeted intake of caffeine should be 240 mg equaling about 400 ml of coffee. However, finishing 400 ml coffee 60 min before exercise is not an easy way to obtain the benefit of caffeine. While this depends on the weight of the individual and one's food preferences, using supplements could be more convenient compared to coffee in this scenario.

### 5.2. Precautions for supplement use

#### 5.2.1. Interaction with medication

It should be noted that nutrients sometimes interact with medications (54–56). Supplements can easily alter the amount of nutrients in the system, which can place an athlete at risk for unintended or insalubrious medication-nutrient interactions. For example, mineral supplements (e.g., calcium, magnesium, or zinc) may blunt the effect of tetracycline on bacterial infections (57). Considering a report that more than 90% of athletes take medications within a 6-month survey period (58), it is likely that an individual elite athlete will need to take one or more medications in their lifetime. Thus, we recommend supplement use only with the confirmation of a sports physician or sports pharmacist who is informed about potential interactions with medications.

#### 5.2.2. Overdose of nutrients or components

Supplements are often good at providing a single nutrient or component; some exist in which one capsule is required to meet or even exceed the recommended dietary allowance. However, the ease of having nutrients and components may cause overdose and subsequent health issues. In fact, a previous investigation confirmed that micronutrient intake in some athletes using supplements exceeds the upper limit (59). In particular, side effects of micronutrient supplements have been frequently reported (60–62). For example, an overdose of vitamin D, which is likely to be deficient among elite athletes (59, 63), including Paralympic athletes (64), has been reported to cause side effects (e.g., vomiting, stomach ache, and appetite reduction) (62). The IOC consensus statement also states the side effects of components such as caffeine and creatine (2). In addition, another previous investigation reported that 86.4% of athletes did not know the side effects of supplements (65). We also confirm the existence of Japanese elite athletes who did not realize the side effects of supplements during our support. Taken together, we emphasize that to avoid overdose and a further reduction in athletes' conditioning, evaluating nutritional status before using supplements, combined with effective nutrition education on supplement use in athletes, is essential.

## 6. Anti-doping

The prevalence of supplement use may raise the risk of health issues and anti-doping violations. Based on a code by the World Anti-Doping Agency (WADA) (66), athletes should understand the meaning of “strict liability,” which is that it is athletes' personal duty to ensure that no prohibited substance enters their bodies. This means that athletes are responsible for any prohibited substances, metabolites, or markers in their samples, regardless of intentionality.

Moreover, prospective cohort data have shown that supplement use in younger generations was related to higher odds of problematic alcohol use and drinking-related risk behaviors at a 7-year follow-up (67). Furthermore, from the perspective of athletes' psychological status, a previous study found that young athletes who used supplements had more positive attitudes toward doping and expressed stronger beliefs that doping is effective compared to non-users (68, 69). Accordingly, we believe supplement use in athletes, particularly adolescents, should be carefully monitored for their long-term health.

### 6.1. Non-labeling of components contamination

The labeling of supplements does not disclose all ingredients because supplements are categorized as conventional foods, not drugs, in Japan and other countries (70). Thus, prohibited substances may exist in supplements (10, 11, 21, 22). Moreover, non-purposed ingredients may contaminate supplements if several products are manufactured at the same facility (9). The non-labeling of components and the potential for contamination are the main reasons we cannot ensure supplements are 100% safe from prohibited substances. For example, a previous investigation in 2021 reported that prohibited substances that were not declared on a label were found in 25 out of 66 products (22). The Japan Anti-Doping Agency (JADA) confirms that supplements that are contaminated by prohibited and/or undeclared substances through the manufacturing process annually (12). At the same time, conventional foods may also be contaminated with prohibited substances. For example, the Institute of Biochemistry and Center of Preventive Doping Research group in Germany detected clenbuterol, a sympathomimetic and anabolic agent, in livestock meat in China (71). The WADA noticed that clenbuterol is utilized as a growth promoter only in China, Mexico, and Guatemala (23). In addition, the WADA introduced countermeasures when growth promoters (clenbuterol, ractopamine, zeranol, zilpaterol, and their metabolites) were found in athletes' urine samples (23).

### 6.2. Anti-doping certificate program and anti-doping strategy

To avoid anti-doping violations as much as possible, the JADA published guidelines in 2019 on a framework for declaring product information on sports supplements (12). In an effort to reduce the risk of anti-doping violations caused by supplement use, the JADA guidelines not only provide a framework for declaring product information but also provide information on the processes required to properly administer this framework (12). The JADA also emphasizes that these guidelines provide risk reduction indices in supplement use and not the insurance of absolute safety (12). Supplement choice that meets the requirements of these guidelines can be effective in lowering the risk of contamination by prohibited substances. The JADA guidelines suggest the following implementations: (1) screening of production facilities, (2) periodic product analysis (more than once a year), (3) disclosure of the results of 1 and 2, and (4) updating and/or deleting disclosed information based on a set of standards (e.g., new results from additional analysis or expiration date of products).

Some third-party organizations (e.g., BSCG, Banned Substances Control Group; NSF, National Sanitation Foundation; LGC, Laboratory of the Government Chemist) provide anti-doping certificate programs that meet the JADA guidelines. Choosing from products with certificate batches by these organizations would be a useful anti-doping strategy for athletes who like to use supplements. The certificate batches indicate that the tested products passed through the program, which determines whether the products include any WADA-prohibited substances. However, there are three precautions regarding certificate batches: (1) there exist third-party organizations that do not meet the JADA guidelines, (2) the certificate batches do not prove 100% safety of products; and (3) the anti-doping certificate program evaluates each product but not an entire company line of products; in the same company, for example, one product may complete the anti-doping certificate program, but other products may not. This can provide a false sense of security for consumers who trust brands but do not necessarily investigate specific products within a brand.

To the best of our knowledge, in Japan, one website introduces products without certificate batches but that are tested by third-party organizations meeting the JADA guidelines (72). Another website in the USA informs of products with prohibited substances (73). We highly recommend a preliminary investigation into the use of products without certificate batches. Depending on the situation, further support from sports pharmacists, sports dietitians, or sports physicians is recommended for the use of products without certificate batches.

However, we cannot ensure the absolute safety of supplements from prohibited substances regardless of the implementation of anti-doping certificate programs. Thus, athletes and support crews should prepare for the possibility that all supplements include prohibited substances, realizing that the responsibility for supplement use is on the athletes themselves. For proof of unintentional doping, we recommend storing approximately 30 g of each supplement (30 g of a sample is typically required for testing in the LGC) with a pouch or pill case until the next doping test or at least recording the lot numbers of products. The recommendation of 30 g depends on the purpose of testing; hence, we recommend seeking preliminary confirmation by the LGC before using supplements. Furthermore, it is also recommended that athletes record when, how much, and for how long they use each kind of supplement.

**Figure 4:**
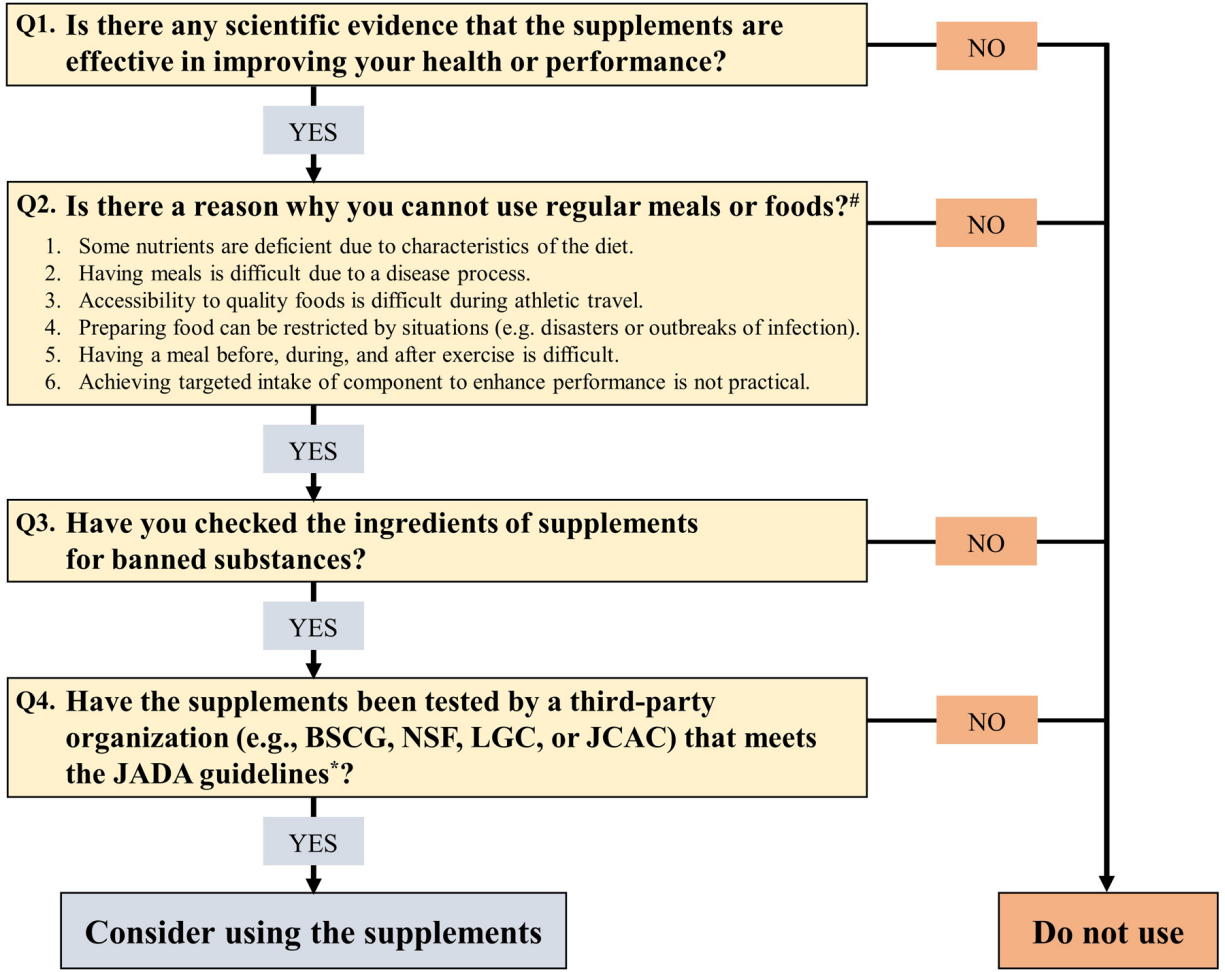
Decision flow chart for supplement use. ^#^The potential reasons for supplement use are presented in Table 1. *The JADA guidelines: (1) screening of production facilities, (2) periodic product analysis (more than once a year), (3) disclosure of the results of 1 and 2, and (4) updating and/or deleting disclosed information based on a set of standards (new results by additional analysis or expiration date of products). BSCG, banned substances control group; NSF, national sanitation foundation; LGC, laboratory of the government chemist; JCAC, Japan chemical analysis center.

These strategies may be useful for reducing penalties (66). However, even if athletes pay attention to anti-doping, take supplements with certificate batches, and confirm that no prohibited substances are on a label, they may be at a disadvantage when anti-doping violations are detected, such as periods of ineligibility and invalidated results of competitions. To inform the choice of supplement use, we created a flow chart (Figure 4) based on information from previous studies (2, 13).

## 7. Strengths and limitations of this study

This review has strengths and limitations. Firstly, to respect the perspectives of athletes and their support networks, we implemented a narrative review. This methodology offers the advantage of addressing practical concerns, such as those involving multiple confounding factors in sports, which are difficult to explore via conventional scientific methodologies. Specifically, elite athletes often confront complex circumstances (e.g., intense training, injury, extensive travel) more frequently than the general population. However, we should acknowledge that our review was not a systematic review; hence, potential bias could exist. This possible bias should be factored into the interpretation of our findings. Subsequently, our study commenced with the provision of support to Japanese athletes who come to the HPSC in Japan. Therefore, our study has the potential to directly benefit Japanese athletes. However, this context should be considered when generalizing our findings to other populations, particularly those from different countries.

## 8. Conclusion

Our study emphasizes the “meal first” approach for improving athletes' health, performance, and anti-doping safety in elite athletes, and the importance of organizing basic factors (e.g., diet, training, and sleep) before considering supplement use, thus maximizing its benefits and safety. Our work can contribute to the education guideline for athletes and their support networks on their nutritional strategy. However, it is also critical to acknowledge the limited evidence advocating the “meal first” approach within the athlete population, likely reflecting a prevalent tendency to prioritize supplements over meals. This highlights an urgent need for continued research and education to rectify misconceptions and promote balanced, effective, and safe nutritional strategies in the world of elite sports.
